# Perceived autonomy support and subjective sport performance evaluation: serial mediation via exercise self-efficacy, autonomous exercise motivation, behavioral engagement, and concentration ability

**DOI:** 10.3389/fpsyg.2026.1826081

**Published:** 2026-05-19

**Authors:** Jingchao Tian, Huiying Zhang, Junjun Sun

**Affiliations:** 1 Department of Physical Education, Dongshin University, Naju, Republic of Korea; 2 Department of Leisure Sports, Kangwon National University, Samcheok, Republic of Korea; 3 School of Foreign Languages, Shandong Vocational and Technical University of International Studies, Rizhao, China

**Keywords:** autonomous exercise motivation, exercise behavioral engagement, exercise concentration ability, exercise self-efficacy, perceived autonomy support, subjective sport performance evaluation

## Abstract

**Objectives:**

Drawing on self-determination theory, self-efficacy theory, and research on exercise-related attentional control, this study examined the mechanism through which perceived autonomy support influences subjective sport performance evaluation, and tested the serial mediating roles of exercise self-efficacy, autonomous exercise motivation, exercise behavioral engagement, and exercise concentration ability.

**Methods:**

A questionnaire survey was administered to adult recreational sport participants, yielding 532 valid responses. The measures included perceived autonomy support, exercise self-efficacy, autonomous exercise motivation, exercise behavioral engagement, exercise concentration ability, and the subjective sport performance evaluation scale. Confirmatory factor analysis and structural equation modeling were conducted using AMOS 26 to evaluate the reliability and validity of the measurement model. Serial mediation effects were tested using bias-corrected bootstrap procedures.

**Results:**

Perceived autonomy support was significantly and positively associated with subjective sport performance evaluation (β = 0.263, SE = 0.057, 95% CI [0.149, 0.378]). Exercise self-efficacy and autonomous exercise motivation constituted a significant serial mediating pathway between perceived autonomy support and subjective sport performance evaluation (β = 0.029, SE = 0.009, 95% CI [0.015, 0.052]). Exercise behavioral engagement and exercise concentration ability also formed a significant serial mediating pathway linking perceived autonomy support to subjective sport performance evaluation (β = 0.025, SE = 0.007, 95% CI [0.013, 0.043]).

**Conclusion:**

Perceived autonomy support was directly associated with adult recreational sport participants’ subjective sport performance evaluation. It was also indirectly related to this evaluation through multiple psychological and behavioral mechanisms. These findings provide theoretical support and practical implications for creating supportive environments in recreational sport contexts.

## 1 Background

Recreational sport participation has become increasingly diverse. As a result, scholars have recognized that objective performance or physical fitness indicators alone cannot fully capture individuals’ lived experiences and psychological states during participation. In contrast, subjective sport performance evaluation reflects individuals’ judgments of their capability enactment, movement execution quality, and overall exercise state. Therefore, it has received growing attention in sport psychology research (Fox and Corbin, 1989; Marsh, 1996). Accordingly, when explaining how such evaluations are formed, researchers have increasingly shifted their focus from single ability- or performance-based accounts to environmentally meaningful factors within exercise contexts.

Self-determination theory posits that perceived autonomy support shapes the quality of motivation and self-regulation. These factors are further related to a range of psychological and behavioral outcomes (Ryan and Deci, 2000). In exercise settings, perceived autonomy support refers to individuals’ experiences of respect and understanding in processes such as decision-making, pace regulation, and goal selection, and is considered an important external condition for fostering positive exercise experiences (Mageau and Vallerand, 2003). Social cognitive theory emphasizes the role of exercise self-efficacy. As a core belief in one’s capabilities, self-efficacy is related to exercise initiation, exercise maintenance, and individuals’ perceptions of their performance (Bandura, 1997). Autonomous exercise motivation has also been regarded as a key psychological variable linking environmental support to exercise experiences, shaping how individuals engage in exercise and the standards they use to evaluate it (Vallerand, 2007).

Beyond these psychological factors, behavioral engagement and cognitive regulation mechanisms during exercise are also critical. Exercise behavioral engagement reflects individuals’ effort, persistence, and attentional involvement in exercise and represents an important pathway through which motivation is translated into actual behavior (Fredricks et al., 2004). Exercise concentration ability refers to the cognitive capacity to sustain attention and inhibit distractions in exercise contexts, which is closely related to execution quality and performance perception (Posner and Rothbart, 2007). Although prior research has identified perceived autonomy support, exercise self-efficacy, autonomous exercise motivation, exercise behavioral engagement, and concentration ability as influential factors, most studies have examined these associations in isolated or partial ways. Among adult recreational sport participants, whether these variables jointly contribute to subjective sport performance evaluation through sequential pathways within a single model remains to be systematically tested with empirical evidence.

### 1.1 Perceived autonomy support and subjective sport performance evaluation (H1)

Perceived autonomy support refers to individuals’ perceptions that significant others or the surrounding environment respect and understand them during activity participation. It also reflects the extent to which their autonomy, personal choice, and self-determination are supported. In recreational sport contexts, such support is typically reflected in participants having decision-making latitude regarding exercise modality selection, goal setting, pace regulation, and strategy or method adjustment. It is also reflected in receiving positive responses and meaningful rationales when needed (Mageau and Vallerand, 2003). Subjective sport performance evaluation refers to adult recreational sport participants’ self-judgments of their exercise performance. These judgments may concern a single exercise bout or a period of exercise. They are based on participants’ subjective experiences of ability use, movement execution quality, and overall exercise state. This evaluation emphasizes individuals’ self-perceptions of the exercise process and outcomes. For example, participants may judge whether they performed to their potential, maintained stable and coordinated movements, experienced a positive exercise state, and were satisfied with their overall performance. This evaluation does not rely solely on objective indicators such as scores, speed, repetitions, or fitness metrics (Fox and Corbin, 1989; Marsh, 1996).

Self-determination theory posits that autonomy support helps satisfy individuals’ basic psychological needs, thereby fostering higher-quality motivation and more adaptive self-regulation, which in turn leads to more favorable psychological and behavioral outcomes (Ryan and Deci, 2000). When adult recreational sport participants perceive a higher level of autonomy support, their exercise participation is more likely to be experienced as self-endorsed rather than compelled. As a result, they may be more likely to develop positive affective experiences and stronger willingness to invest effort, and to interpret their exercise performance using standards that are more conducive to personal growth. Moreover, autonomy support may reduce evaluative anxiety, perceived external control, and feelings of frustration, enabling individuals to focus more on task completion and process improvement. This shift may ultimately enhance their subjective judgments of whether their performance was adequately enacted, whether their movements were stable, and whether their overall state was optimal.

Based on these theoretical considerations and proposed mechanisms, perceived autonomy support is expected to be directly associated with adult recreational sport participants’ subjective evaluation of their own sport performance. Therefore, H1 is proposed: Perceived autonomy support is positively associated with subjective sport performance evaluation.

### 1.2 Perceived autonomy support → exercise self-efficacy → autonomous exercise motivation → subjective sport performance evaluation (H2)

In recreational sport participation, perceived autonomy support represents a key contextual characteristic. It emphasizes respect for individuals’ choices, encouragement of self-initiated decision-making, and reduction of controlling interference. Such supportive experiences may strengthen individuals’ sense of control and agency in exercise. They may also help participants view exercise as a self-directed action rather than a passive task driven by external pressure. This may be associated with more positive judgments about their own capabilities (Koka and Hein, 2005). In addition, an autonomy-supportive environment may shape how individuals interpret performance-related information. Under high autonomy support, individuals are more likely to attribute improvement and success to their own effort and competence development, which in turn reinforces confidence in their abilities and facilitates the formation of more stable exercise self-efficacy. Exercise self-efficacy refers to individuals’ subjective judgments about their capability to accomplish exercise tasks and cope with exercise-related challenges, and it constitutes an important psychological foundation for regulating exercise behavior and maintaining sustained participation (Hagger et al., 2003; Standage et al., 2005; Teixeira et al., 2012).

Autonomous exercise motivation refers to a form of motivation in which individuals engage in exercise based on intrinsic interest, personal valuing, or self-endorsed goals. Its core features are self-determination and internal endorsement of the behavior. Autonomous motivation is not determined unidirectionally by the external context; rather, it develops through a gradual process of internalization as individuals’ competence beliefs and perceived behavioral control become strengthened. From this perspective, exercise self-efficacy provides a critical cognitive basis for the development of autonomous exercise motivation. When individuals have stronger confidence in themselves, they are more likely to experience exercise participation as controllable and meaningful, which enhances their spontaneous willingness to engage and their tendency to regulate exercise behavior in accordance with self-set goals and standards (Chase, 2001). Exercise self-efficacy may also be related to motivational internalization. This may occur through individuals’ affective responses to exercise processes and outcomes. Individuals with higher self-efficacy are more likely to experience a sense of mastery and accomplishment when facing challenges, and such positive experiences are considered important psychological sources of intrinsic motivation, thereby making exercise behavior more readily internalized as part of one’s self-values (Biddle et al., 1999).

Increases in autonomous exercise motivation can further influence individuals’ evaluative standards and reference frames for judging exercise performance, thereby relating to subjective sport performance evaluation. Subjective sport performance evaluation refers to individuals’ comprehensive self-judgments of their ability enactment, movement execution quality, and overall state during or after exercise, based on their subjective experiences and self-perceptions. When exercise behavior is driven by highly autonomous motivation, individuals are more likely to develop adaptive self-regulation experiences and to make more favorable psychological evaluations of their behavioral outcomes (Hagger and Chatzisarantis, 2007). From a behavioral regulation perspective, when autonomous regulation is high, individuals tend to use personal improvement, effort investment, and perceived skill mastery as key evaluation criteria rather than relying solely on external outcomes or social comparison (Wilson et al., 2003), which makes them more likely to form positive and stable subjective sport performance evaluation. This mechanism may be particularly salient in recreational sport contexts: individuals with higher autonomous motivation are more likely to experience accomplishment and satisfaction during exercise and integrate these positive experiences into their overall judgments of their sport performance (Teixeira et al., 2012). When exercise behavior aligns closely with individuals’ internal values, post-exercise self-reflection is more likely to generate a positive self-evaluative structure that is less dependent on short-term performance fluctuations and more grounded in holistic perceptions of the engagement process, thereby strengthening the positivity of subjective sport performance evaluation (Pelletier et al., 2013).

Taken together, H2 is proposed: Perceived autonomy support is indirectly and positively associated with subjective sport performance evaluation through the serial mediating roles of exercise self-efficacy and autonomous exercise motivation.

### 1.3 Perceived autonomy support → exercise behavioral engagement → exercise concentration ability → subjective sport performance evaluation (H3)

Exercise behavioral engagement refers to individuals’ effort, persistence, and proactive attentional involvement during exercise participation. It reflects how motivation is translated into actual behavior. Perceived autonomy support is an important social-contextual characteristic. It provides choice, respects individuals’ preferences, and reduces controlling interference. In this context, individuals may be more likely to interpret exercise as a self-chosen action. This reduces resistance and psychological pressure, allowing more psychological resources to be invested in the exercise task itself, which is reflected in greater effort intensity and more stable persistence. Empirical evidence indicates that higher autonomy support is associated with stronger behavioral persistence and engagement (Edmunds et al., 2007). When instructors adopt autonomy-supportive practices—such as supporting choice, providing explanatory feedback, and reducing controlling language—participants’ initiative and sustained engagement increase significantly (Cheon et al., 2012). Autonomy support has also shown stable associations with active participation in physical education and recreational exercise contexts (Hagger et al., 2005). Accordingly, the present study posits that higher perceived autonomy support facilitates greater exercise behavioral engagement.

Exercise concentration ability refers to individuals’ capacity to allocate, sustain, and regulate attention during exercise. It is reflected in sustained focus on task-relevant information and inhibition of irrelevant distractions. This ability is not determined solely by innate traits; rather, it can be gradually developed and strengthened through sustained participation and repeated practice. From a behavioral perspective, higher exercise behavioral engagement provides necessary conditions for the training and consolidation of attentional control: when individuals continuously invest effort and maintain focus during exercise, they are more likely to develop stable task orientation and to optimize attentional allocation strategies through repetition (Ericsson et al., 1993). Attentional control theory suggests that sustained engagement enables the executive system to regulate attentional resources more effectively, enhancing processing of key information while inhibiting distracting stimuli (Eysenck et al., 2007). Individuals who are more highly engaged in training or practice are better able to maintain attentional focus and performance under complex tasks and distracting conditions (Beilock and Carr, 2001). Repeatedly coping with tasks that concurrently impose motor and attentional demands under high engagement may further improve attentional allocation and control efficiency (Gopher et al., 1989). Therefore, this study proposes that exercise behavioral engagement is positively associated with exercise concentration ability.

Improvements in exercise concentration ability may subsequently influence how individuals extract, integrate, and utilize information and feedback during exercise, thereby relating to subjective sport performance evaluation. Individuals with stronger concentration ability can focus more consistently on critical movement cues and bodily feedback during exercise, which facilitates clearer perceptions of movement execution quality and overall state, leading to more consistent and more positive subjective judgments. In contrast, when attention is easily disrupted, perceptions of movement execution tend to be fragmented, and subjective evaluations are more susceptible to situational factors and emotional fluctuations. Stable attentional focus helps individuals develop clearer cognitions of movement quality, thereby influencing the consistency of self-evaluation (Wulf et al., 2010). Reviews of attentional expertise in sport similarly suggest that stronger attentional control is closely related to more effective performance monitoring and higher-quality execution processes (Memmert, 2009). In addition, attentional control is linked to error monitoring and self-correction: when attentional resources are allocated more efficiently, individuals are more likely to detect deviations in a timely manner and adjust accordingly, and these immediate feedback and correction experiences may contribute to more positive performance perceptions (Abernethy et al., 2007).

Taken together, H3 is proposed: Perceived autonomy support is indirectly and positively associated with subjective sport performance evaluation through the serial mediating roles of exercise behavioral engagement and exercise concentration ability.

### 1.4 Research model and hypotheses

**FIGURE 1:**
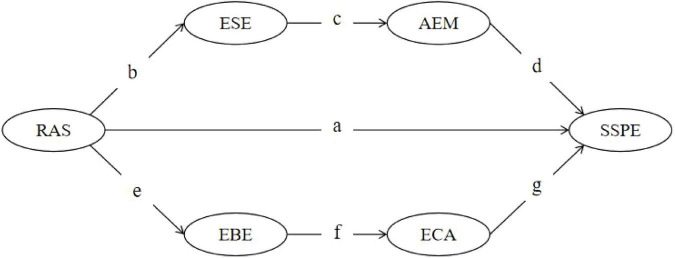
Serial mediation structural model. PAS, perceived autonomy support; ESE, exercise self-efficacy; AEM, autonomous exercise motivation; EBE, exercise behavioral engagement; ECA, exercise concentration ability; SSPE, subjective sport performance evaluation. Direct effect “a”: PAS → SSPE; effect “bcd”: PAS → ESE → AEM → SSPE; effect “efg”: PAS → EBE → ECA → SSPE.

The present study developed the research model shown in Figure 1. The model was grounded in self-determination theory, self-efficacy theory, and prior research on exercise-related attentional control. It also integrated theoretical logic and empirical evidence from previous studies. This model systematically illustrates the mechanisms through which perceived autonomy support is associated with subjective sport performance evaluation via multiple psychological and behavioral pathways. Specifically, in addition to examining the direct effect of perceived autonomy support on subjective sport performance evaluation, the model further tests the serial mediating roles of exercise self-efficacy, autonomous exercise motivation, exercise behavioral engagement, and exercise concentration ability.

More specifically, the present study proposes a dual serial mediation model because perceived autonomy support may operate through two theoretically distinct but complementary mechanisms: a competence-based pathway (PAS → ESE → AEM → SSPE) and a behavioral-attentional pathway (PAS → EBE → ECA → SSPE). Compared with a single-mediator or simple parallel-mediation model, this specification better captures the sequential nature of psychological internalization and behavioral regulation while still allowing for a direct association between PAS and SSPE. First, perceived autonomy support may be positively associated with individuals’ exercise self-efficacy, which in turn is related to autonomous exercise motivation and ultimately to subjective sport performance evaluation. Second, perceived autonomy support may be positively associated with individuals’ exercise behavioral engagement, which may further be related to exercise concentration ability and, consequently, to subjective sport performance evaluation. Based on the foregoing theoretical analysis, the following hypotheses are proposed:

**H1:** Perceived autonomy support is positively associated with subjective sport performance evaluation (path “a”).

**H2:** Perceived autonomy support is indirectly associated with subjective sport performance evaluation through the serial mediating effects of exercise self-efficacy (path “b”) and autonomous exercise motivation (path “c”), leading to subjective sport performance evaluation (path “d”), that is, a “b–c–d” serial mediation effect.

**H3:** Perceived autonomy support is indirectly associated with subjective sport performance evaluation through the serial mediating effects of exercise behavioral engagement (path “e”) and exercise concentration ability (path “f”), leading to subjective sport performance evaluation (path “g”), that is, an “e–f–g” serial mediation effect.

## 2 Materials and methods

### 2.1 Sample size calculation

This study adopted a descriptive cross-sectional design. Sample size estimation was based on the Leslie Kish formula, which is suitable for estimating a single proportion. The formula is as follows:


$$n=\frac{Z^{2}\,p\,\left(1-p\right)}{d^{2}}$$

In this formula, *Z* is the critical value of the standard normal distribution corresponding to a 95% confidence level (Z = 1.96). p denotes the expected proportion; in the absence of prior information, it was conservatively set to 0.50. *d* represents the allowable margin of error, which was set to 0.043 in the present study. This margin of error was chosen to obtain a relatively precise proportion estimate under feasible survey costs. It also provided a more stable basis for subsequent SEM parameter estimation and met the minimum sample size requirement. Substituting these parameters into the formula yielded a theoretical minimum sample size of approximately 520. Potential invalid responses and data quality control were considered during questionnaire collection. Ultimately, 532 valid responses were retained. This sample size met the requirements for subsequent statistical analyses and structural equation modeling.

### 2.2 Participants

This study recruited adult recreational sport participants using convenience sampling. The questionnaire was distributed and collected online. Therefore, the sample may have been more likely to include individuals with internet access, willingness to participate in online surveys, and existing interest or involvement in recreational sport activities. During data collection, quality control procedures were implemented to exclude responses that were clearly careless or exhibited invalid response patterns. A total of 532 valid questionnaires were retained (*N* = 532).

Regarding participants’ basic characteristics, the gender distribution was relatively balanced, with 270 men (50.75%) and 262 women (49.25%). The mean age was 33.67 years (SD = 9.283). In terms of education, 273 participants (51.32%) had an educational level below a bachelor’s degree, and 259 (48.68%) held a bachelor’s degree or above. For occupation, the largest group was corporate employees (general staff) (*n* = 194, 36.47%), followed by professionals/technical personnel (*n* = 90, 16.92%), self-employed/freelancers (*n* = 71, 13.35%), civil servants/public institution staff (*n* = 61, 11.47%), service industry workers (e.g., catering, retail, culture and tourism; *n* = 59, 11.09%), students (undergraduate/master’s/doctoral; *n* = 43, 8.08%), and retirees (*n* = 14, 2.63%).

With respect to recreational sport participation characteristics, the primary activity types included running/walking (*n* = 145, 27.26%), yoga/Pilates (*n* = 111, 20.86%), gym-based exercise (*n* = 84, 15.79%), ball sports (*n* = 68, 12.78%), swimming (*n* = 64, 12.03%), and outdoor trail running/off-road activities (*n* = 60, 11.28%). In terms of participation frequency, 269 participants (50.56%) exercised once per week or less, 115 (21.62%) exercised 2–3 times per week, and 148 (27.82%) exercised three times per week or more. For session duration, 272 participants (51.13%) reported exercising for less than 30 min, 97 (18.23%) for 30–60 min, and 163 (30.64%) for more than 60 min per session. Regarding the duration of continued participation in their current primary recreational sport activity, 155 participants (29.14%) reported less than 1 year, 269 (50.56%) reported 1–3 years of participation, and 108 (20.30%) reported more than 3 years of participation. In addition, 181 participants (34.02%) engaged in recreational sport in a fixed venue or organized setting, whereas 351 (65.98%) participated mainly individually or in non-fixed formats.

### 2.3 Measures

All measurement instruments used in this study were adapted from well-established scales that had been validated in prior research. Because the survey was administered to Chinese adult recreational sport participants, the questionnaire was presented in Chinese. For scales originally available in English, a translation and back-translation procedure was conducted to ensure linguistic equivalence. First, two bilingual researchers independently translated the original English items into Chinese. Second, another bilingual researcher, who was not involved in the initial translation, back-translated the Chinese version into English. The research team then compared the back-translated version with the original items and resolved discrepancies through discussion.

To ensure cultural and contextual appropriateness, the wording of several items was further adapted to fit the context of adult recreational sport participation rather than competitive sport or school-based physical education. For example, references to “coach,” “teacher,” “training,” or “sport performance” were adjusted when necessary to reflect recreational exercise settings, exercise instructors, relevant organizations, or participants’ self-evaluations of recent exercise performance. These modifications did not change the conceptual meaning of the original constructs.

All items were rated on a 7-point Likert scale (1 = strongly disagree, 7 = strongly agree). Scale internal consistency was examined using SPSS. The results showed that Cronbach’s α coefficients ranged from 0.924 to 0.945, indicating good internal consistency reliability across the measures.

#### 2.3.1 Perceived autonomy support (PAS)

The perceived autonomy support scale was adapted from Standage et al. (2005) and contained 12 items assessing participants’ subjective perceptions of autonomy support provided by coaches or relevant organizations during exercise/training. The items covered key autonomy-supportive behaviors, including: providing multiple options and autonomy, understanding participants’ ideas and feelings, enabling open communication, expressing confidence in participants’ ability and performance, encouraging questions and responding patiently and thoroughly, clearly explaining goals and required tasks, appropriately addressing emotional reactions, respecting individual feelings, encouraging proactive decision-making, eliciting participants’ views before introducing new training methods, and listening to how participants prefer to train/exercise.

#### 2.3.2 Exercise self-efficacy (ESE)

Exercise self-efficacy was measured using the health-context self-efficacy scale developed by Schwarzer and Renner (2009), with wording revised to reflect exercise participation characteristics. The scale comprised 10 items evaluating individuals’ confidence in maintaining an exercise plan, managing time, establishing habits, adjusting strategies, and achieving goals. It emphasized perceived capability to sustain exercise behavior even when fatigued, busy, in a poor mood, or facing difficulties.

#### 2.3.3 Autonomous exercise motivation (AEM)

Autonomous exercise motivation was assessed using the scale developed by Dubnjakovic (2016) based on a self-determination theory framework, including 13 items. It measured the extent to which individuals participate in exercise for autonomous reasons such as value endorsement (e.g., exercise benefits health, fitness, and long-term development), interest and learning experiences (e.g., learning new skills and mastering techniques), and positive affective experiences (e.g., satisfaction with improvement, enjoyment of challenge, sense of accomplishment, excitement/pleasure, and relaxation).

#### 2.3.4 Exercise behavioral engagement (EBE)

Exercise behavioral engagement was measured using an eight-item scale adapted from Scanlan et al.’s (1993) sport commitment model and tailored to recreational sport contexts. The scale assessed time investment and effort in current exercise participation, willingness to continue, prioritizing exercise activities, and proactive time management behaviors to ensure ongoing participation.

#### 2.3.5 Exercise concentration ability (ECA)

Exercise concentration ability was measured with an eight-item scale adapted from the measurement approach proposed by Smith et al. (2006) and revised for the exercise context. It assessed participants’ ability to sustain concentration during exercise, direct attention to current movements/tasks, inhibit external distractions, remain focused under high intensity or pressure, and rapidly refocus after attention has been disrupted.

#### 2.3.6 Subjective sport performance evaluation (SSPE)

Subjective sport performance evaluation was assessed using the 12-item scale developed and validated by Lee et al. (2023), with minor wording adjustments for recreational sport contexts. The scale measured participants’ satisfaction with recent exercise performance, perceived alignment with self-expectations, perceived ability enactment and task completion quality, maintenance of performance under fatigue, pressure, or challenging conditions, and subjective judgments regarding others’ recognition.

### 2.4 Data analysis

Data analysis in this study proceeded in three stages: data preprocessing, measurement model testing, and structural model testing. First, SPSS was used to organize and screen the returned questionnaire data. After removing invalid cases, 532 valid questionnaires were retained. Before model estimation, descriptive statistics were calculated for all study variables, including means, standard deviations, skewness, and kurtosis. Correlation analyses were also conducted to examine preliminary associations. These analyses provided a basis for subsequent structural equation modeling.

Second, CFA was performed in AMOS 26 to evaluate the measurement model, including the structural validity and measurement adequacy of each latent construct. Model fit was assessed using multiple indices, including χ^2^/*df*, GFI, CFI, NFI, TLI, and RMSEA. In addition, standardized factor loadings, SMC, CR, and AVE were used to examine convergent validity and internal consistency. Discriminant validity was evaluated by comparing the square root of each construct’s AVE with the corresponding inter-construct correlations. If localized misfit was observed, standardized residuals and modification indices were consulted to identify potentially problematic items. Item removal was not based solely on statistical criteria. Each item was also reviewed for theoretical relevance, conceptual overlap with retained items, and contextual appropriateness for adult recreational sport participation. Items were removed only when they showed localized misfit or redundancy and when their exclusion did not alter the theoretical meaning or dimensional coverage of the corresponding construct.

Finally, after establishing an acceptable measurement model, an SEM was specified to test the direct path from perceived autonomy support to subjective sport performance evaluation, as well as two serial mediation pathways (PAS → ESE → AEM → SSPE; PAS → EBE → ECA → SSPE). Mediation effects were tested using bias-corrected bootstrap procedures with a specified number of resamples (e.g., 5,000), and 95% confidence intervals were computed for indirect effects. An indirect effect was considered statistically significant when its confidence interval did not include zero. Direct effects, indirect effects, and total effects were reported, and the study hypotheses were evaluated based on overall model fit and the significance of path coefficients.

## 3 Results

### 3.1 Common method bias and model fit

The data were collected at a single time point using self-report questionnaires. Therefore, the present study examined CMB and evaluated its potential influence on the relationships among variables. Following commonly recommended procedural remedies, the survey was administered anonymously, participants were informed that there were no right or wrong answers, evaluative pressure was minimized, and items were presented in a randomized order to reduce social desirability and same-source bias. Subsequently, CMB was further assessed at the statistical level to ensure that the data quality was adequate for subsequent structural model testing.

Regarding model fit, AMOS 26 was used to evaluate the overall fit of the proposed model, and the results are presented in Table 1. The fit indices showed that χ^2^ = 1787.982, *df* = 1532, and χ^2^/*df* = 1.167, which is below the commonly recommended cutoff value of 3. The GFI was 0.900, meeting the acceptable threshold of 0.90. In addition, the CFI = 0.988, NFI = 0.924, and TLI = 0.988 all exceeded the recommended value of 0.90. The RMSEA was 0.018, which is well below the criterion of 0.06. Taken together, these results indicate that the proposed model demonstrated a good overall fit to the sample data. Therefore, the measurement and structural models adequately represented the observed data, providing a sound basis for the subsequent structural path analysis and serial mediation testing.

**TABLE 1:** Model fit.

χ ^2^	*df*	χ ^2^/*df*	GFI	CFI	NFI	TLI	RMSEA
1787.982	1532	1.167 < 3	0.900 ≥ 0.90	0.988 ≥ 0.90	0.924 ≥ 0.90	0.988 ≥ 0.90	0.018 < 0.06

### 3.2 Confirmatory factor analysis

Confirmatory factor analysis was conducted in AMOS 26 for all latent variables. This analysis examined the structural validity and reliability of the measurement instruments. Following the recommendations of Fornell and Larcker (1981), and informed by standardized residuals and modification indices, items with relatively low contributions to model fit or apparent redundancy were reviewed. The decision to remove items was based on both statistical and theoretical considerations. Specifically, PAS2, PAS10, ESE3, AEM1, AEM9, and SSPE4 were removed. These items showed localized misfit and overlapped conceptually with retained items within the same constructs. PAS2 and PAS10 overlapped with retained autonomy-support items concerning choice, respect, and communication; ESE3 overlapped with retained self-efficacy items related to confidence in maintaining exercise under barriers; AEM1 and AEM9 overlapped with retained autonomous motivation items reflecting personal value, interest, enjoyment, and perceived benefits; and SSPE4 overlapped with retained subjective performance evaluation items concerning satisfaction, task completion, and perceived ability enactment. Thus, item deletion reduced redundancy. It did not change the theoretical meaning or dimensional coverage of the latent constructs. The CFA results for the final model are presented in Table 2.

**TABLE 2:** Confirmatory factor analysis.

Variable	Item	Significance test parameters				Std.	SMC	CR	AVE
		Unstd.	SE	*Z*	*P*				
PAS	PAS1	1	–	–	–	0.980	0.961	0.946	0.637
	PAS3	0.796	0.027	28.783	***	0.797	0.637		
	PAS4	0.752	0.028	26.618	***	0.772	0.596		
	PAS5	0.827	0.027	29.844	***	0.809	0.655		
	PAS6	0.751	0.028	26.178	***	0.766	0.588		
	PAS7	0.795	0.029	26.707	***	0.773	0.598		
	PAS8	0.792	0.029	26.840	***	0.775	0.601		
	PAS9	0.736	0.029	25.429	***	0.756	0.573		
	PAS11	0.767	0.028	26.587	***	0.771	0.596		
	PAS12	0.726	0.028	25.182	***	0.753	0.567		
ESE	ESE1	1	–	–	–	0.986	0.972	0.940	0.637
	ESE2	0.773	0.030	25.811	***	0.759	0.576		
	ESE4	0.780	0.028	27.548	***	0.781	0.61		
	ESE5	0.751	0.029	25.252	***	0.751	0.565		
	ESE6	0.789	0.028	27.383	***	0.779	0.607		
	ESE7	0.766	0.028	26.933	***	0.773	0.598		
	ESE8	0.763	0.028	26.884	***	0.773	0.598		
	ESE9	0.791	0.029	27.158	***	0.776	0.603		
	ESE10	0.767	0.028	26.999	***	0.774	0.600		
AEM	AEM2	1	–	–	–	0.763	0.582	0.935	0.568
	AEM3	0.971	0.053	18.070	***	0.749	0.561		
	AEM4	0.913	0.051	17.849	***	0.741	0.550		
	AEM5	0.977	0.053	18.298	***	0.757	0.573		
	AEM6	0.965	0.052	18.575	***	0.767	0.588		
	AEM7	0.958	0.053	17.783	***	0.738	0.546		
	AEM8	0.919	0.051	17.902	***	0.743	0.552		
	AEM10	0.998	0.053	18.558	***	0.766	0.587		
	AEM11	0.971	0.053	18.220	***	0.754	0.569		
	AEM12	1.001	0.053	18.619	***	0.768	0.591		
	AEM13	0.942	0.052	17.802	***	0.739	0.547		
EBE	EBE1	1	–	–	–	0.976	0.954	0.929	0.624
	EBE2	0.744	0.030	24.289	***	0.746	0.557		
	EBE3	0.757	0.029	25.766	***	0.767	0.589		
	EBE4	0.757	0.030	25.053	***	0.757	0.573		
	EBE5	0.760	0.029	26.132	***	0.772	0.596		
	EBE6	0.741	0.029	24.921	***	0.755	0.571		
	EBE7	0.756	0.030	24.984	***	0.756	0.572		
	EBE8	0.759	0.029	25.480	***	0.763	0.582		
ECA	ECA1	1	–	–	–	0.991	0.982	0.926	0.612
	ECA2	0.771	0.030	25.299	***	0.751	0.565		
	ECA3	0.756	0.029	25.347	***	0.752	0.566		
	ECA4	0.695	0.029	23.833	***	0.730	0.534		
	ECA5	0.756	0.030	25.091	***	0.748	0.561		
	ECA6	0.777	0.029	25.974	***	0.760	0.579		
	ECA7	0.730	0.028	25.385	***	0.752	0.567		
	ECA8	0.742	0.030	24.213	***	0.736	0.542		
SSPE	SSPE1	1	–	–	–	0.985	0.971	0.939	0.586
	SSPE2	0.781	0.031	24.495	***	0.741	0.549		
	SSPE3	0.741	0.030	24.181	***	0.736	0.543		
	SSPE5	0.781	0.030	25.385	***	0.753	0.568		
	SSPE6	0.745	0.031	24.041	***	0.734	0.539		
	SSPE7	0.791	0.031	24.992	***	0.748	0.560		
	SSPE8	0.747	0.031	23.726	***	0.729	0.533		
	SSPE9	0.766	0.031	24.664	***	0.743	0.553		
	SSPE10	0.756	0.031	24.148	***	0.736	0.542		
	SSPE11	0.765	0.029	25.623	***	0.756	0.573		
	SSPE12	0.758	0.033	22.867	***	0.716	0.513		

***P < 0.001; PAS, perceived autonomy support; ESE, exercise self-efficacy; AEM, autonomous exercise motivation; EBE, exercise behavioral engagement; ECA, exercise concentration ability; SSPE, subjective sport performance evaluation.

The results showed that the standardized factor loadings of the observed indicators ranged from 0.716 to 0.991, and all loadings were statistically significant (*p* < 0.001), indicating that the indicators adequately represented their corresponding latent constructs and that the relationships between latent variables and observed indicators were strong.

Further analyses showed that the CR values for the latent constructs ranged from 0.926 to 0.946. These values exceeded the recommended threshold of 0.70, suggesting good internal consistency and measurement stability.

In addition, the AVE values ranged from 0.568 to 0.637, all above the criterion of 0.50, indicating that a substantial proportion of the variance in the indicators was explained by their respective latent constructs and that the measurement model demonstrated good convergent validity.

Overall, the measurement model exhibited satisfactory structural validity and reliability, providing a robust measurement foundation for subsequent structural equation modeling and hypothesis testing.

### 3.3 Correlation and descriptive statistical analyses

Before formal hypothesis testing, descriptive statistics and Pearson correlation analyses were conducted for the main variables. The results are presented in Table 3. The means of the variables ranged from 4.271 to 4.576, and the standard deviations ranged from 1.447 to 1.693, indicating that overall levels were in the middle-to-upper range of the scales. Skewness values ranged from −0.079 to 0.048, and kurtosis values ranged from −1.458 to −1.317, suggesting that the distributions showed no pronounced skewness or extreme kurtosis and met the basic assumptions for subsequent structural equation modeling.

**TABLE 3:** Descriptive statistics and correlations.

Variable	PAS	ESE	AEM	EBE	ECA	SSPE
PAS	**0.798**	–	–	–	–	–
ESE	0.340***	**0.798**	–	–	–	–
AEM	0.397***	0.321***	**0.754**	–	–	–
EBE	0.264***	0.299***	0.335***	**0.790**	–	–
ECA	0.365***	0.362***	0.355***	0.323***	**0.782**	–
SSPE	0.354***	0.326***	0.367***	0.301***	0.360***	**0.766**
*M*	4.576	4.346	4.271	4.332	4.451	4.564
SD	1.693	1.680	1.447	1.622	1.655	1.580
Skew	−0.079	−0.076	−0.027	−0.060	0.048	−0.071
Kurtosis	−1.458	−1.410	−1.317	−1.341	−1.435	−1.389
AVE	0.637	0.637	0.568	0.624	0.612	0.586

***P < 0.001; The bold italic values represent the square roots of AVE. PAS, perceived autonomy support; ESE, exercise self-efficacy; AEM, autonomous exercise motivation; EBE, exercise behavioral engagement; ECA, exercise concentration ability; SSPE, subjective sport performance evaluation.

The correlation results indicated that PAS was significantly and positively correlated with ESE, AEM, EBE, ECA, and SSPE (*r* = 0.264–0.397, *p* < 0.001). These findings suggest that higher perceived autonomy support is associated with higher levels of self-efficacy, motivational quality, behavioral engagement, attentional regulation, and subjective sport performance evaluation.

In addition, based on the criterion proposed by Miao and Zhang (2024), the square roots of AVE for all latent variables (bold italics on the diagonal of Table 3) were greater than the correlations with other constructs, supporting adequate discriminant validity among the constructs.

### 3.4 Serial mediation effect analysis

**FIGURE 2:**
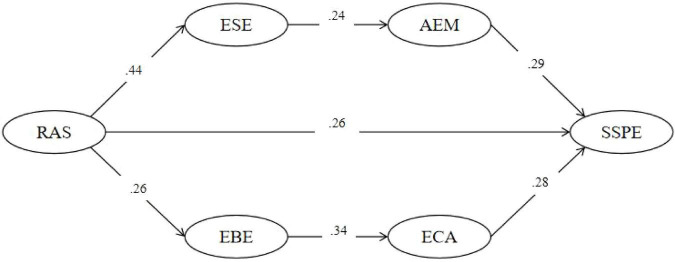
Serial mediation path model. PAS, perceived autonomy support; ESE, exercise self-efficacy; AEM, autonomous exercise motivation; EBE, exercise behavioral engagement; ECA, exercise concentration ability; SSPE, subjective sport performance evaluation. Direct effect “a”: PAS → SSPE; effect “bcd”: PAS → ESE → AEM → SSPE; effect “efg”: PAS → EBE → ECA → SSPE.

To examine the mechanism through which PAS is associated with SSPE, a structural equation model was specified after establishing an acceptable measurement model. Two serial mediation pathways were tested using bias-corrected bootstrap procedures (5,000 resamples, 95% confidence intervals). The standardized path coefficients of the structural model are shown in Figure 2, and the results of the serial mediation tests are presented in Table 4.

**TABLE 4:** Serial mediation effect analysis.

Hypothesis and path	β	Product of coefficients		95% CI
		SE	*Z*	
H1: Path “a”	0.263**	0.057	4.614	[0.149, 0.378]
H2: Path “bcd”	0.029***	0.009	3.222	[0.015, 0.052]
H3: Path “efg”	0.025***	0.007	3.571	[0.013, 0.043]
Total	0.318**	0.055	5.782	[0.212, 0.432]

**P < 0.01; ***p < 0.001; direct effect “a”: PAS → SSPE; effect “bcd”: PAS → ESE → AEM → SSPE; Effect “efg”: PAS → EBE → ECA → SSPE .

The results indicated that the direct effect of perceived autonomy support on subjective sport performance evaluation (path “a”) was significantly positive (β = 0.263, SE = 0.057, 95% CI [0.149, 0.378]). This suggests that, after controlling for the mediators, perceived autonomy support remained significantly associated with individuals’ subjective sport performance evaluation, thereby supporting H1.

Regarding indirect effects, the serial pathway PAS → ESE → AEM → SSPE (paths “b–c–d”) was significantly positive (β = 0.029, SE = 0.009, 95% CI [0.015, 0.052]). This finding indicates that perceived autonomy support was positively associated with subjective sport performance evaluation through higher levels of ESE, which were further associated with higher levels of AEM, thus supporting H2.

Similarly, the serial pathway PAS → EBE → ECA → SSPE (paths “e–f–g”) was significantly positive (β = 0.025, SE = 0.007, 95% CI [0.013, 0.043]). This result suggests that perceived autonomy support was associated with subjective sport performance evaluation through higher levels of EBE, which were further associated with higher levels of ECA, thereby supporting H3.

Overall, the total effect of perceived autonomy support on subjective sport performance evaluation was significant (Total β = 0.318, SE = 0.055, 95% CI [0.212, 0.432]). Taken together, these findings indicate that perceived autonomy support not only exerts a significant direct effect, but also operates indirectly through two sequential transmission mechanisms—self-efficacy → autonomous motivation and behavioral engagement → concentration ability—providing further support for the proposed dual serial mediation model.

## 4 Discussion

This study focused on adult recreational sport participants and examined the mechanisms through which perceived autonomy support (PAS) is associated with subjective sport performance evaluation (SSPE). Drawing on self-determination theory, self-efficacy theory, and perspectives on attentional control, the results showed that PAS was directly associated with SSPE and was also indirectly associated with SSPE through two sequential pathways: exercise self-efficacy (ESE) → autonomous exercise motivation (AEM) and exercise behavioral engagement (EBE) → exercise concentration ability (ECA). These findings suggest that SSPE in recreational sport contexts may be shaped by social contextual support, competence-related beliefs, motivational quality, behavioral engagement, and attentional regulation.

### 4.1 Positive association between PAS and SSPE (H1 supported)

From a social-contextual perspective, an autonomy-supportive environment may be directly associated with more positive exercise experiences. When participants feel respected, understood, and able to make meaningful choices, they may experience less pressure and greater self-congruence. Such experiences may help them focus more on movement execution and bodily feedback, thereby supporting more favorable subjective sport performance evaluation (Baard et al., 2004; Gagné and Deci, 2005).

Autonomy support may also be related to participants’ evaluative reference points. In autonomy-supportive contexts, individuals are more likely to focus on the task process and movement quality rather than external evaluation or outcome comparison (Reeve, 2012). This may be especially relevant in recreational sport, where performance evaluation often lacks a unified objective benchmark and relies more heavily on process experience. Therefore, perceived autonomy support may remain directly associated with more favorable SSPE, even when mediating mechanisms are considered (Adie et al., 2012).

### 4.2 PAS → ESE → AEM → SSPE (H2 supported)

The findings showed that perceived autonomy support was indirectly associated with SSPE through the serial mediating pathway of ESE → AEM, thereby supporting H2. This suggests that the association between autonomy support and performance perception may involve a sequential psychological process. Specifically, autonomy support may be related to stronger competence beliefs, which are further associated with more autonomous forms of exercise motivation.

More specifically, PAS was first associated with higher exercise self-efficacy. As a central judgment of one’s action capabilities, self-efficacy is related to individuals’ engagement, persistence, and interpretation of exercise outcomes. When individuals develop stronger competence beliefs, they may view exercise tasks as more controllable and worthy of sustained effort (Maddux, 1995). Building on this foundation, self-efficacy may be related to more autonomous exercise motivation because individuals with stronger competence beliefs are more likely to integrate exercise goals into their self-system (Carver and Scheier, 2001; Sheldon and Elliot, 1999).

Autonomous exercise motivation may further be associated with SSPE by shaping evaluative standards. Individuals with higher autonomous motivation are more likely to evaluate behavioral outcomes using internal criteria, such as effort investment, perceived skill mastery, and process experience, rather than relying on external outcomes or social comparison (Milyavskaya et al., 2015). For adult recreational sport participants, this internalized evaluative framework may be particularly important because performance judgments often depend more on process experience and self-regulatory cues than on formal competitive outcomes. Thus, the ESE → AEM pathway helps explain how PAS is related to more favorable SSPE among recreational sport participants.

### 4.3 PAS → EBE → ECA → SSPE (H3 supported)

The results also supported the serial mediating pathway of EBE → ECA between PAS and SSPE, thereby supporting H3. This finding extends prior accounts that primarily emphasized motivation and competence beliefs by highlighting a behavioral engagement–cognitive regulation pathway. It suggests that autonomy support is related not only to individuals’ willingness to exercise, but also to how they invest effort and regulate attention during exercise.

Within this serial mechanism, PAS was first associated with higher exercise behavioral engagement. Behavioral engagement reflects effort intensity, persistence, and prioritization of the task. When the exercise context provides greater choice and psychological respect, participants may experience exercise as more self-endorsed and may invest time and energy more consistently in movement execution and pace regulation (Kanfer, 1990). Such engagement may provide an important behavioral foundation for concentration and attentional regulation.

Higher EBE may then be related to stronger ECA. Executive attention theory suggests that sustained engagement helps individuals maintain stable attention to task-relevant information while inhibiting task-irrelevant distractions (Engle, 2002). Empirical work also indicates that persistent behavioral experiences are associated with attentional control performance, including attentional stability and task-switching efficiency (Pessoa et al., 2003). When individuals have stronger concentration ability, they may monitor movement cues and bodily feedback more clearly and consistently. This may support more stable and favorable post-exercise performance judgments (Beilock and Carr, 2001).

The potential influence of sport type should also be considered. The present sample included participants involved in different recreational activities, such as running/walking, yoga/Pilates, gym-based exercise, ball sports, swimming, and outdoor trail running/off-road activities. These activities may involve different attentional demands and cognitive requirements. For instance, yoga/Pilates may require sustained internal attention to posture and breathing, whereas ball sports may require rapid attention shifting, decision-making, and responses to external cues. Therefore, the association between behavioral engagement, concentration ability, and SSPE may vary across sport types. Future studies should use multi-group analyses or sport-specific samples to examine whether the proposed model operates similarly across different exercise modalities.

## 5. Limitations

Several limitations and directions for future research should be acknowledged. This study focused on adult recreational sport participants. It examined two serial pathways linking perceived autonomy support to subjective sport performance evaluation: self-efficacy → autonomous motivation and behavioral engagement → attentional control. First, the study employed a cross-sectional self-report design. Although the structural model revealed significant associations among the proposed pathways, the cross-sectional design does not allow for strict causal inference or conclusions about temporal ordering. Future studies could use longitudinal designs or experimental manipulations of autonomy-supportive contexts to test temporal ordering and causal direction. Second, all variables were derived from the same source, and although common method bias was addressed, self-report measures may still inflate associations among constructs. Future work is encouraged to incorporate multi-source data and objective indicators, such as training logs, movement quality ratings, or reports from others, to provide convergent validation. Third, the study used convenience sampling and online data collection, which may have introduced selection bias. In particular, individuals who were already physically active, interested in exercise, or willing to participate in online sport-related surveys may have been overrepresented. As a result, the findings should be generalized with caution to less physically active adults, non-regular exercisers, or populations with limited access to online surveys. Moreover, because the sample consisted of general recreational sport participants, caution is also needed when applying the findings to specific sports or organized training settings, particularly because different sport types may involve different attentional demands, cognitive requirements, feedback structures, and environmental complexity. Future studies should use more diverse recruitment strategies and include participants with different physical activity levels to further examine the generalizability of the proposed model.

## 6 Conclusion

Based on self-determination theory, self-efficacy theory, and research on attentional control, this study systematically examined the mechanisms linking perceived autonomy support to adult recreational sport participants’ subjective sport performance evaluation. The findings indicate that perceived autonomy support is directly associated with individuals’ subjective evaluations of their own exercise performance. It is also indirectly related to such evaluations through two complementary serial pathways. First, perceived autonomy support is associated with higher autonomous exercise motivation through higher exercise self-efficacy, which is further related to more favorable subjective sport performance evaluation. Second, perceived autonomy support is associated with higher exercise behavioral engagement, which is further related to stronger exercise concentration ability and, ultimately, to more favorable subjective sport performance evaluation. Collectively, these findings suggest that adult recreational sport participants’ performance perceptions are not shaped by a single psychological or behavioral factor. Instead, they may reflect the joint role of social contextual support, competence-related beliefs, motivational quality, behavioral engagement, and cognitive regulation processes.

At the theoretical level, this study extends the framework of perceived autonomy support mechanisms from a dual serial mediation perspective by integrating the self-efficacy–motivation pathway and the behavioral engagement–attentional control pathway within a single model. It enriches explanations of how subjective sport performance evaluation is formed in recreational sport contexts and provides new theoretical evidence for understanding how supportive environments shape exercise experiences through sequential psychological–behavioral–cognitive processes.

At the practical level, the findings suggest several actionable strategies for recreational sport organizations, fitness centers, and exercise instructors. First, instructors should provide participants with meaningful exercise choices, explain the purpose of training tasks, and encourage self-directed goal setting. These practices may strengthen participants’ perceived autonomy support and improve their exercise experiences. Second, instructors should use specific encouragement and constructive feedback to help participants recognize progress and build confidence. This may enhance exercise self-efficacy and support more autonomous exercise motivation. Third, programs should include clear participation plans, progressive exercise tasks, and brief attention-focusing cues during practice. These strategies may increase behavioral engagement, improve concentration, and support more positive subjective sport performance evaluations.

## Data Availability

The datasets presented in this study can be found in online repositories. The names of the repository/repositories and accession number(s) can be found in the article/Supplementary material.

## Supplementary material

Supplementary Table 1 Detailed sample data used in the analysis.
